# Antiviral defense systems in the rumen microbiome

**DOI:** 10.1128/msystems.01521-24

**Published:** 2025-01-14

**Authors:** Johan S. Sáenz, Bibiana Rios-Galicia, Jana Seifert

**Affiliations:** 1 Institute of Animal Science, University of Hohenheim, Stuttgart, Germany; 2 HoLMiR—Hohenheim Center for Livestock Microbiome Research, University of Hohenheim, Stuttgart, Germany; University of Connecticut, Storrs, Connecticut, USA

**Keywords:** defense mechanism, phages, rumen microbiome, CRISPR-Cas

## Abstract

The continuous interaction between phages and their respective hosts has resulted in the evolution of multiple bacterial immune mechanisms. However, the diversity and prevalence of antiviral defense systems in complex communities are still unknown. We therefore investigated the diversity and abundance of viral defense systems in 3,038 high-quality bacterial and archaeal genomes from the rumen. In total, 14,241 defense systems and 31,948 antiviral-related genes were identified. Those genes represented 114 unique system types grouped into 49 families. We observed a high prevalence of defense systems in the genomes. However, the number of defense systems, defense system families, and system density varied widely from genome to genome. Additionally, the number of defense system per genome correlated positively with the number of defense system families and the genome size. Restriction modification, Abi, and cas system families were the most common, but many rare systems were present in only 1% of the genomes. Antiviral defense systems are prevalent and diverse in the rumen, but only a few are dominant, indicating that most systems are rarely present. However, the collection of systems throughout the rumen may represent a pool of mechanisms that can be shared by different members of the community and modulate the phage–host interaction.

**IMPORTANCE:**

Phages may act antagonistically at the cell level but have a mutualistic interaction at the microbiome level. This interaction shapes the structure of microbial communities and is mainly driven by the defense mechanism. However, the diversity of such mechanism is larger than previously thought. Because of that, we described the abundance and diversity of the antiviral defense system of a collection of genomes, metagenome-assembled genomes (MAGs) and isolates, from the rumen. While defense mechanisms seem to be prevalent among bacteria and archaea, only a few were common. This suggests that most of these defense mechanisms are not present in many rumen microbes but could be shared among different members of the microbial community. This is consistent with the “pan-immune system” model, which appears to be common across different environments.

## INTRODUCTION

It is estimated that the demand for meat and milk will rise between 70% and 80% of the current production by 2050 (1). Part of the current needs are met by raising cattle, which account for almost one third of all domesticated ruminants (2). However, sustainable beef and dairy production is challenged by economic and political issues, environmental factors, feed efficiency, and animal and human health (3, 4). Recent studies have shown that some of these problems are linked to the rumen microbiome.

The massive use of sequencing technologies has shown that the rumen is a complex ecosystem consisting of bacteria, archaea, protozoa, fungi, and viruses (5–7). This group of organisms contributes to the metabolism of complex plant materials and the production of volatile fatty acids (VFAs), which provide most of the energy required by the animal. Rumen bacteria and archaea have been associated with feed efficiency, methane production, animal health, and fat and protein content of milk (8–11). Similarly, it has been shown that anaerobic fungi play a crucial role in the efficient degradation of fiber through enzymatic and physical processes (12). Rumen viruses are diverse and organism specific, carry diverse auxiliary metabolic genes, and have the potential to modulate nutrient recycling, fiber degradation, and methanogenesis (13–15). Based on metagenomic surveys, the rumen seems to harbor a larger proportion of lytic phages compared with other environments, which can lead to a higher selective pressure for the prokaryotic community (16, 17). However, the lysogenic capacity of the rumen virome needs to be corroborated by microscopy and culture-based methods. In addition, dietary changes or perturbations can modulate viral richness and diversity, which has been shown to affect microbial metabolism (15).

Phages shape bacterial and archaeal populations and are a reservoir of accessory genes that facilitate the mobility of genetic material (18). The continuous interaction between phages and their respective hosts has resulted in the evolution of bacterial immune mechanism and phage counter-defense strategies (19, 20). This arms race is a major driving force of molecular innovation and genetic diversification. Defense mechanism in prokaryotes are diverse and can be divided between innate and adaptive immune systems (21). The prevalence of defense mechanisms ranges from zero to several systems between cells. Defense systems can be compose by single or multiple genes, mainly associated with nucleases, helicases, proteases, kinases, ATPases, and reverse transcriptases (22, 23). Antiviral defense mechanism are classify mainly in three groups: nucleic acid degradation, abortive infection, and inhibition of DNA/RNA synthesis, but the mechanism of the majority of systems remains unknown (24). Moreover, the effective immunity against phages seems to be driven by within-cell (25), presence of single or multiple system, and pan-genome dynamics (21). Previous studies have shown that rumen bacteria harbor multiple CRISPR-Cas and RM systems (14, 26), which can be associated with the modulation of phage–host interaction. However, a detail description of the diversity and prevalence of antiviral defense systems in the rumen is lacking. We explored the prevalence and diversity of antiviral defense mechanisms in 3038 high quality genomes from the rumen. This study shows that the repertoire of defense mechanisms in the rumen is broad and that the pan-immunity is mainly dominated by innate immune mechanisms.

## MATERIALS AND METHODS

### Data collection and data set description

In total 5,990 genomes from the rumen microbiome were collected for the present study. Briefly, 5,588 metagenomes assembled genomes (MAGs) from the MGnify (https://www.ebi.ac.uk/about/news/updates-from-data-resources/cow-rumen-catalogue-v10-released-mgnify/) cow rumen v1.0 MAG catalogue and 402 genomes of isolates from the Hungate 1000 catalogue of reference genomes were obtained (5). The MAG data set is based on data collected from European cattle (27) and African cattle (28).

### Quality control of MAGs

CheckM2 (29) was used to estimate the quality of the genomes, and only the high-quality genomes with >90% completeness and <5% contamination were kept for further analysis. Additionally, the N50, coding density, gene length, genome size and GC content of the genomes were calculated using CheckM2. The genomes were taxonomically classified using GTDB-tk v2.1 (30) and the GTDB database R207_v2 (31).

### Antiviral defense prediction

Antiviral defense mechanisms were identified from all genomes using PADLOC v1.1 (23) and the PADLOC-DB. Systems labeled as other due to incomplete detection of essential genes were discarded. Defense systems were grouped by families using the PADLOC-DB metadata (https://github.com/padlocbio/padloc-db/blob/master/sys_meta.txt) and the references to relevant literature (https://github.com/padlocbio/padloc-db/blob/master/system_info.md). The density of defense systems per genome was calculated as the total defense systems per genome divided by the genome size (kbp). CRISPR-Cas genes and arrays were detected using CRISPRCasTyper v1.6 (32). All predicted CRISPR spacers from complete or incomplete CRISPR-Cas arrays were kept. Viral genomes from the rumen virome database (RVD) were matched against CRISPR spacers using Blast (33), only those with >95% identity, ≤1 mismatch and <0.0004 e-value were considered spacer to protospacer matches. Host-virus interaction network showing the spacer-protospacer match was created using Cytoscape (34).

### Prophage detection and identification

Collected genomes were searched for prophages using Virsorter2 v2.2.3 (35). Only contigs longer than >10,000 were used. All contigs identified as prophages were kept for further analysis. Prophages were quality checked using checkV (36). Prophages with 0 viral genes and >1 host gene were discarded and prophages with 0 viral genes and 0 host genes were kept. A prophage was considered complete if it was predicted as such by checkV.

### Phylogenetic placement

Phylogenetic tree of the whole 3038 genomes was created using Phylophlan (37) and 400 universal marker genes, with --medium diversity and --accurate options. Phylogenetic relation among species of *Butyrivibrio*, Ca. *Limimorpha*, *Prevotella* and *Ruminococcus* was calculated using a subset of genes present in at least 90% of all genomes (core genes) for each genus. *Butyrivibrio*, *Prevotella* and *Ruminococcus* were selected due to their key role in the rumen and high prevalence of defense systems, while Ca. *Limimorpha* due to its novelty. Core genes were extracted, aligned, and concatenated using the tool concatenated-gene-alignment-fasta from Anvi’o v 7.1 (38). The extracted genes (55 of *Butyrivibrio*, 71 of *Limimorpha*, 13 of *Prevotella* and 46 of *Ruminococcus*) were used to observe phylogenetic relatedness. Phylogenetic trees were inferred by maximum-likelihood using the concatenated genes of each genome with FastTree2 (39). Taxonomic information, number of defense families and numbers of prophages were displayed in a phylogenetic tree using iTOL (40).

### Data wrangling and data availability

All data wrangling and statistical analysis were performed in R v4.1.2 (41) and the packages “tidyverse” (42), “data.table,” “pheatmap,” “ggdist,” “patchwork,” “ggtext,” and “viridis” were used. Medians across groups were compared using the nonparametric Kruskal-Wallis test (kruskall.test function). Additionally, correlations were calculated using the Spearman’s rank correlation coefficient (cor.test, method = “spearman”). Data processing workflow as well as other R scripts for data wrangling and visualization can be found as a Jupyter-lab notebook at https://github.com/SebasSaenz/Papers_wf. The MGnify cow rumen v1.0 MAG catalogue collection can be found at http://ftp.ebi.ac.uk/pub/databases/metagenomics/mgnify_genomes/cow-rumen/v1.0/, and the Hungate 1000 catalogue of reference genomes from the rumen microbiome is available at https://genome.jgi.doe.gov/portal/TheHunmicrobiome/TheHunmicrobiome.info.html.

## RESULTS

### Data set description

A total of 5,990 rumen microbiome genomes were collected from the MGnify genome resource (43) and the Hungate1000 collection (Table S1). In summary, 5,578 of the collected genomes were metagenome-assembled genomes (MAGs) and 412 were bacterial and archaeal isolates. After checking the quality of the assemblies using CheckM2, 2,930 genomes were classified as high-quality drafts, 3,035 were classified as medium quality drafts, and zero were classified as low-quality drafts. The remaining genomes were discarded because they had more than 10% contamination or completeness could not be calculated. Based on genome quality, 3,038 genomes with ≥90% completeness were retained for further analysis. Briefly, the selected genomes had a mean 107,308 bp ± 274,286 N50, 0.901 ± 0.0263 coding density, 337 bp ± 27.8 gene length, 2,535,744 bp ± 794,061 genome size and 48.5% ± 8.6% GC content (Table S2). In terms of taxonomic classification, 62 genomes were classified as *Archaea* by GTDB-TK (30), while 2,976 were classified as *Bacteria*. In addition, 22 phyla were identified across the data set, of which 10 were represented by more than 20 genomes. Genomes classified as *Bacillota* were the most abundant in the data set representing 50.8% of the total genomes, followed by *Bacteroidota* (32.4%), *Pseudomonadota* (3.7%), *Actinomycetota* (3.4%), *Cyanobacteriota* (2.5%) and *Methanobacteriota* (1.7%). Moreover, genomes identified as the genus *Prevotella* (8.4%) were the most prevalent, followed by *Cryptobacteroides* (6.6%), uncultured CAG-791 (3.3%), *Ruminococcus* group E (3.0%) and *Sodaliphilus* (2.9%).

### Prevalence of defense systems

**Fig 1:**
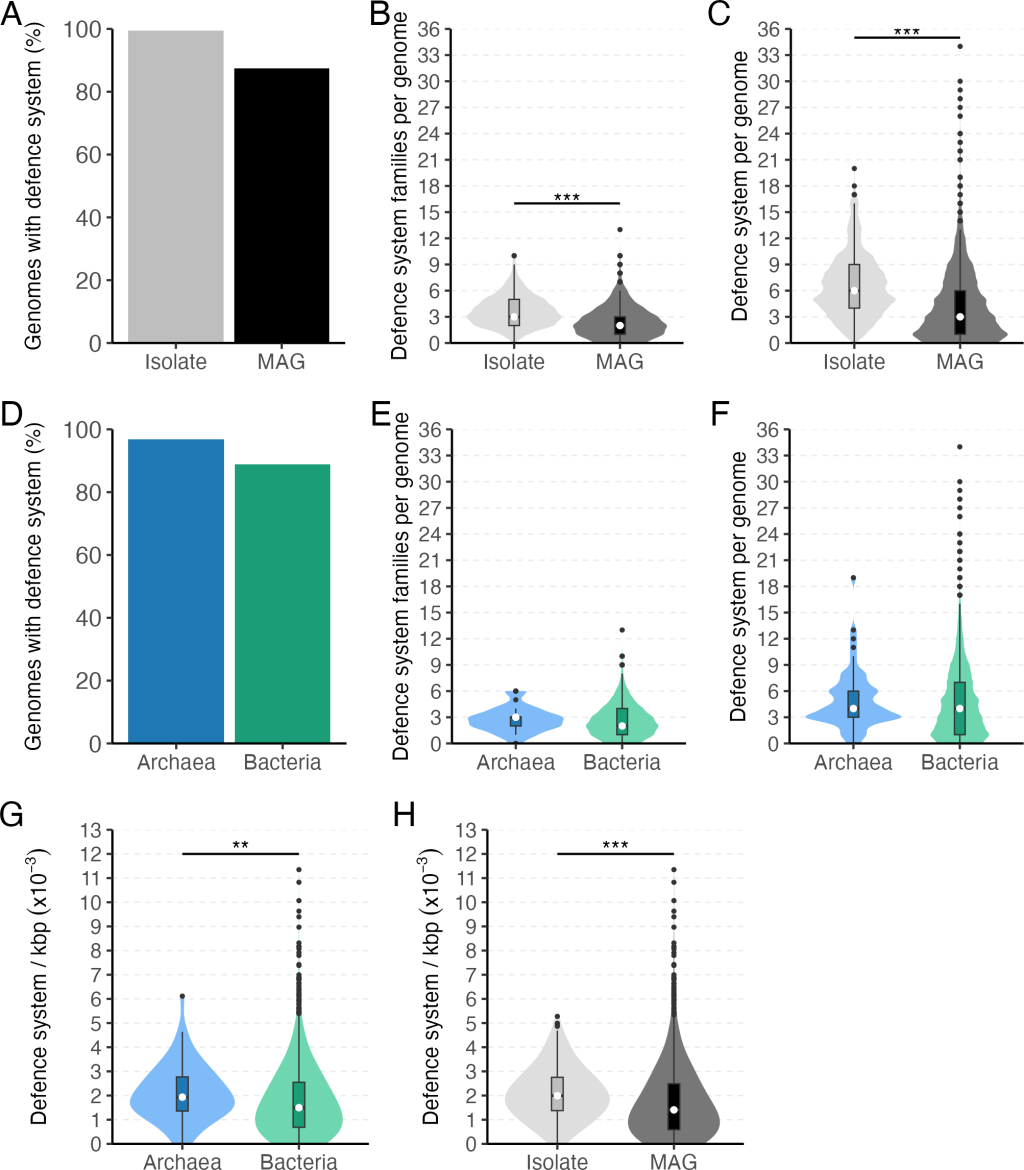
Prevalence of defense systems in the rumen. (**A**) The percentage of genomes with at least one defense system, (**B**) the number of defense systems per genome, (**C**) the number of unique defense system families per genome in isolates and MAGs, (**D**) the percentage of genomes with at least one defense system, (**E**) the number of defense systems per genome, and (**F**) the number of unique defense system families per genome in *Archaea* and *Bacteria*. (G and H) The defense system density (per genome per kb) in (G) *Archaea* and *Bacteria* and (H) isolate and MAGs.

**Fig 2:**
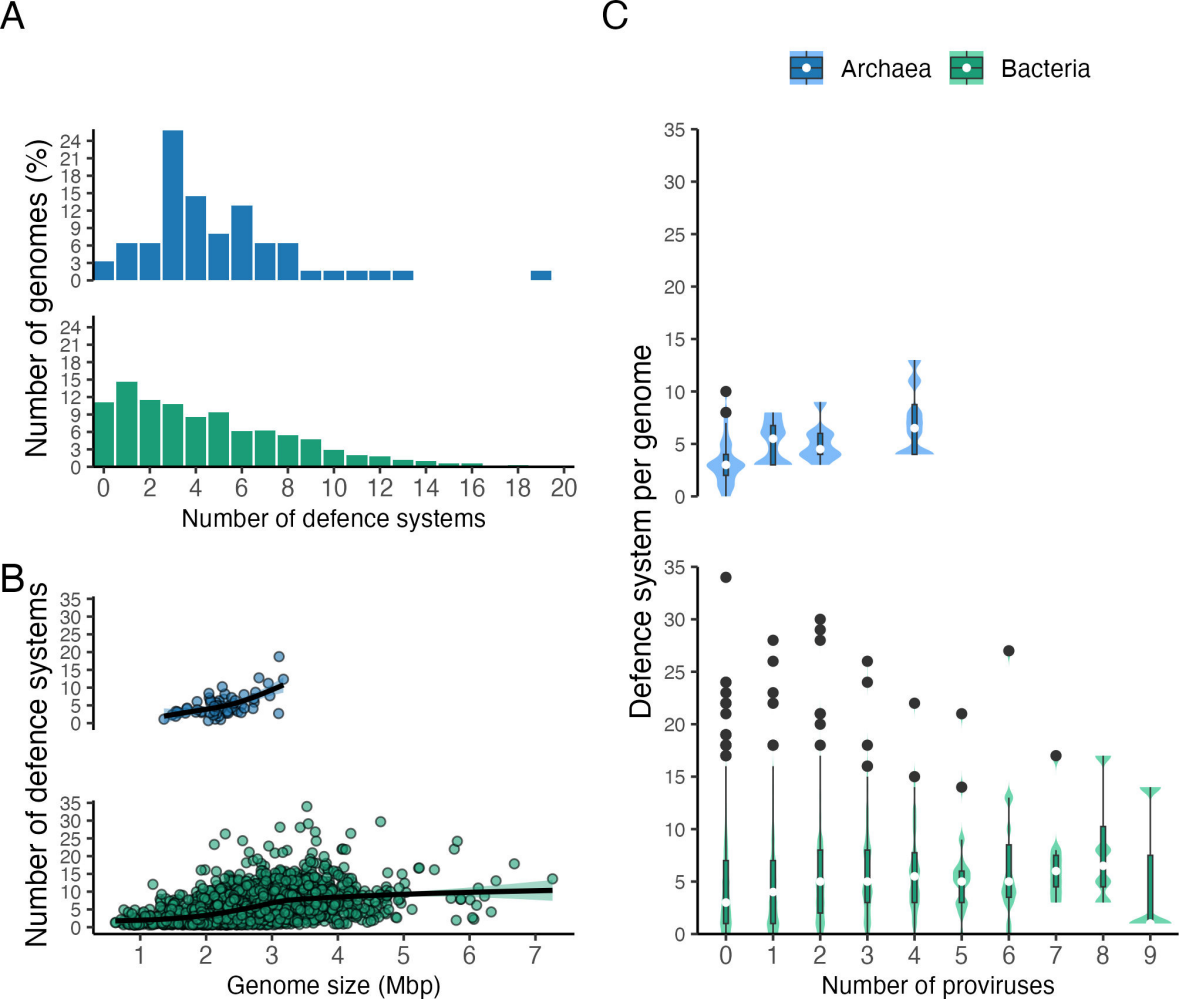
Distribution of the total number of defense systems per genome and their correlation with genomes size and prophage prevalence. (**A**) The percentage of genomes carrying different number of defense systems, (**B**) correlation between the number of defense system per genome and the genome size, and (C) correlation between the number of defense system per genome and the number of proviruses per genome.

Antiviral defense systems are prevalent in the rumen microbiome. In total, 14,241 defense systems and 31,948 antiviral related genes, representing 114 unique system types, were identified across all genomes. Out of the 3,038 genomes, 89% of them carried at least one antiviral defense system. Approximately, 99% of isolates harbored at least one system, compared to 87% of the MAGs (Fig. 1A). On average, isolates had a larger genome (Fig. S1), number of defense system families (*P*-value < 2.2e−16; Fig. 1B), number of defense systems (*P*-value < 2.2e−16; Fig. 1C) and defense systems density (*P*-value = 1.051e−15; Fig. 1H) compared with the MAGs. Similarly, 96% of archaeal genomes harbored at least one defense system, compared to 88% of bacterial genomes (Fig. 1D; Table S3). On average, *Archaea* seem to carry 4.9 systems per genome while *Bacteria* 4.7 (*P*-value = 0.1875; Fig. 1F). However, this number highly varies, being zero the minimum (3.2% archaeal and 11.1% bacterial genomes) up to 34 in the Ca. *Limimorpha*. Approximately 92% of archaeal and bacterial genomes carry between one to ten systems but the remaining genomes harbor between 11 to 34 and 11 to 19 systems in *Bacteria* and *Archaea*, respectively (Fig. 2A). The density of defense systems (per genome and per kb) significantly differed between *Archaea* and *Bacteria* (*P*-value = 0.003752; Fig. 1G), 2.12 × 10^−3^ and 1.75 × 10^−3^, respectively. Additionally, after grouping the systems into families we found that the average number of unique antiviral defense families in archaeal and bacterial genomes is similar, 2.8 and 2.6 respectively (*P*-value = 0.1456; Fig. 1E). The minimum number of families found was zero and 13 the maximum in the uncultured *Lachnospiraceae* CAG-603.

When compared by phyla, between 78% and 100% of the genomes carry at least one defense system (Fig. S2), being *Verrucomicrobiota* and *Fibrobacterota* the highest and *Pseudomonadota* the lowest. *Bacillota* and *Bacteroidota* are among the most prevalent phyla in the data set, with 84% and 96% of the genomes exhibiting defensive systems respectively. Similarly, on average *Verrucomicrobiota* and *Fibrobacterota* genomes have the highest number of defense systems and defense system density (Fig. S2).

We then used Virsorter2 to detect proviral sequences longer than 10,000 bp in the genomes and CheckV to evaluate their quality. The results were compared with the number of defense systems found in each genome. Forty percent of the genomes harbored at least one provirus, being zero the lowest and 10 the highest in one bacterial genome. A positive correlation was observed between the average number of defense systems and the number of proviruses per genome in archaea (Spearman ⍴ = 0.575, *P*-value = 1.005e−06; Fig. 2C), however this was not observed in the bacterial genomes (Spearman ⍴ = 0.080, *P*-value = 1.04e−05). In contrast, the genome size positively correlates with the number of defense systems in archaeal (Spearman ⍴ = 0.528, *P*-value = 1.421e−05) and bacterial genomes (Spearman ⍴ = 0.525, *P*-value < 2.2e−16; Fig. 2B). The relation between the number of proviruses and the genome size has been previously evaluated as potential drivers of the number of antiviral systems in prokaryotic genomes and plasmids (24, 44).

### Diversity and abundance of defense systems

**Fig 3:**
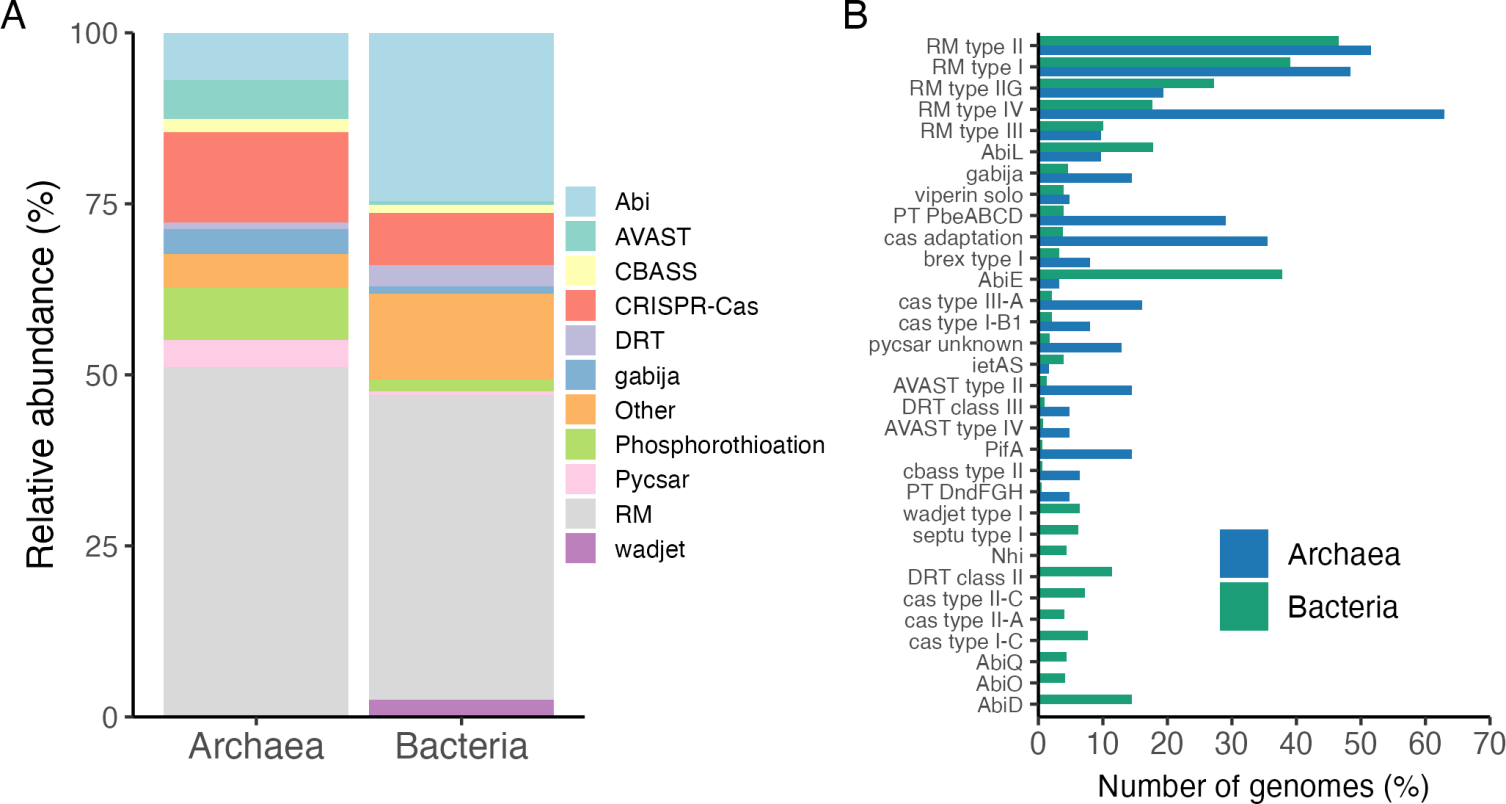
Abundance and diversity of defense systems within *Bacteria* and *Archaea* in the rumen. (**A**) The relative abundance of all defense systems grouped by families in *Bacteria* and *Archaea* and (B) the percentage of genomes per defense system. Defense system families with a relative abundance <1.5% were grouped in the “other” category. The defense systems that were found in <2% of genomes are not shown.

To estimate the diversity of antiviral defense systems, all predicted systems were grouped into families using the metadata provided by PADLOC-DB. In total, 114 different system types were found across all genomes, and they were grouped in 49 families. All of them were found in bacterial genomes, whereas only 16 of them were found in the archaeal genomes. RM, Abi, and cas system families were present in 72.1%, 55.2%, and 27.9% of all genomes. Moreover, 28 of the families were present only in 1% of the genomes. In terms of relative abundance, systems belonging to RM were the most abundant in *Archaea* (51.1%) and *Bacteria* (44.5%) compared to the total systems found (Fig. 3A and B). However, not always the most abundant systems were equally present in both groups. For example, the abundance of the Abi system was 6.9% and 24.6% in *Archaea* and *Bacteria*, respectively. Similarly, the AVAST family represented 5.6% and 0.4% of all systems found in *Archaea* and *Bacteria*, respectively. Additionally, the relative abundance of 34 of the total families was less than 1%.

### Defense systems in key members of the rumen

**Fig 4:**
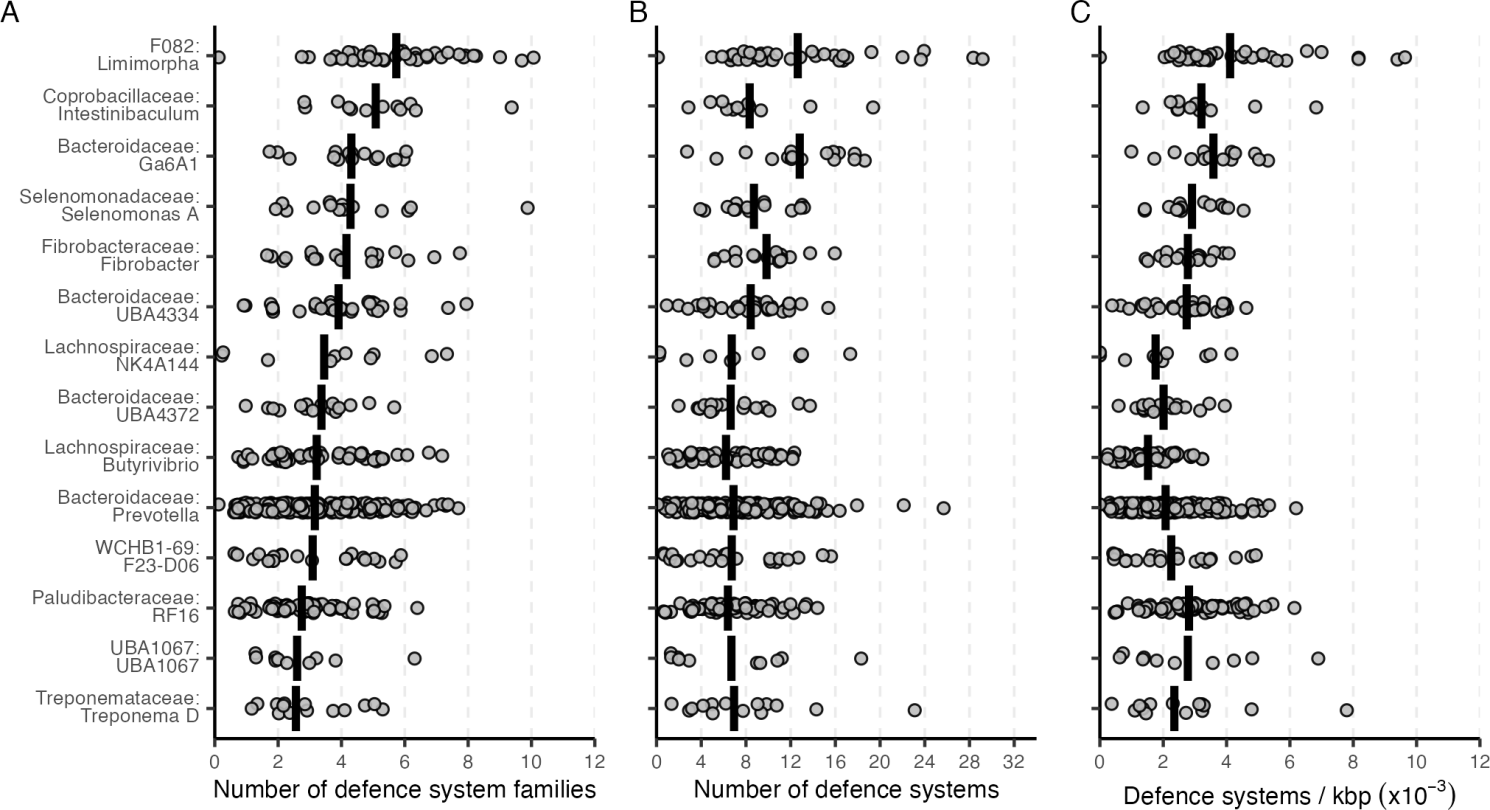
Number of defense systems in different genera from the rumen. (**A**) Number of defense system families, (**B**) number of defense systems and (C) defense system density (per genome per kbp) found in the top 14 genera with the highest average number of defense systems per genome. The vertical bar represents the average number of defense systems and system families. Genera with at least 10 genomes are shown. The labels in the *x*-axis represent the family:genus.

To corroborate if the antiviral defense systems are enriched in a specific taxon, we selected all genera represented by more than 10 genomes (*n* = 57). The number of defense systems and families of the selected genera were compared. Both total numbers of systems and number of different families vary between genera. On average, these genomes (genera represented by at least 10 genomes) carried 4.6 defense systems and 2.6 defense system families. The uncultivated genus Ga6A1 (12.8) and Ca. *Limimorpha* (12.6) had the highest number of systems, followed by *Fibrobacter* (9.8) (Fig. 4B). Similar trends were observed when the number of families and defense system density were compared (Fig. 4A and C). None of the genera were totally devoid of defense systems but *Enterousia* and several uncultivated bacteria had a low prevalence of systems. Additionally, we observed a positive correlation between the number of system and families, which indicates that *Bacteria* or *Archaea* carrying several defense systems will also harbor more defense system families (Fig. S3).

**Fig 5:**
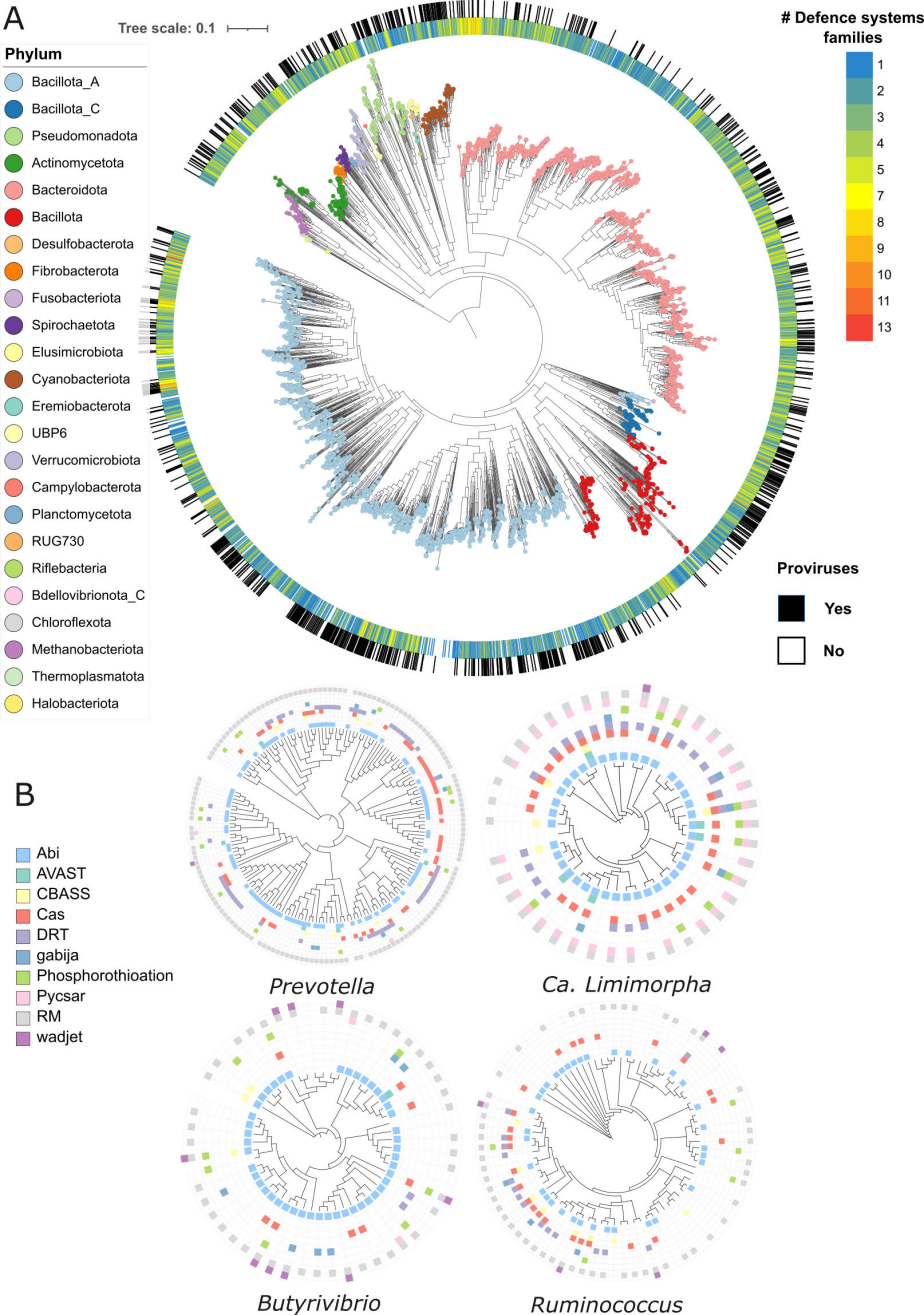
Distribution of defense systems across the rumen prokaryotic community. (**A**) Phylogenetic tree of the rumen microbiome (3038 genomes) based on 400 universal markers. (**B**) Phylogenetic tree based on core genes of four members of the rumen microbiome: *Prevotella*, Ca. *Limimorpha*, *Butyrivibrio* and *Ruminococcus*.

Additionally, the diversity of defense system and system families varies across the dominant members of the rumen (Fig. 5A). For example, between 37 and 97% of genomes of the genera *Prevotella*, Ca. *Limimorpha*, *Butyrivibrio,* and *Ruminococcus* carry RM and Abi systems (Fig. 5B). However, 22, 31, 24, and 23 different defense system families are present in less than 20% of the same genomes, respectively. The Ca. *Limimorpha* seems to be an example of a genus carrying a core group of defense genes, because at least 50% of its genomes harbored RM, Abi, Pycsar, cas, and DRT systems (Fig. 5B).

### CRISPR-Cas arrays and phage interaction

**Fig 6:**
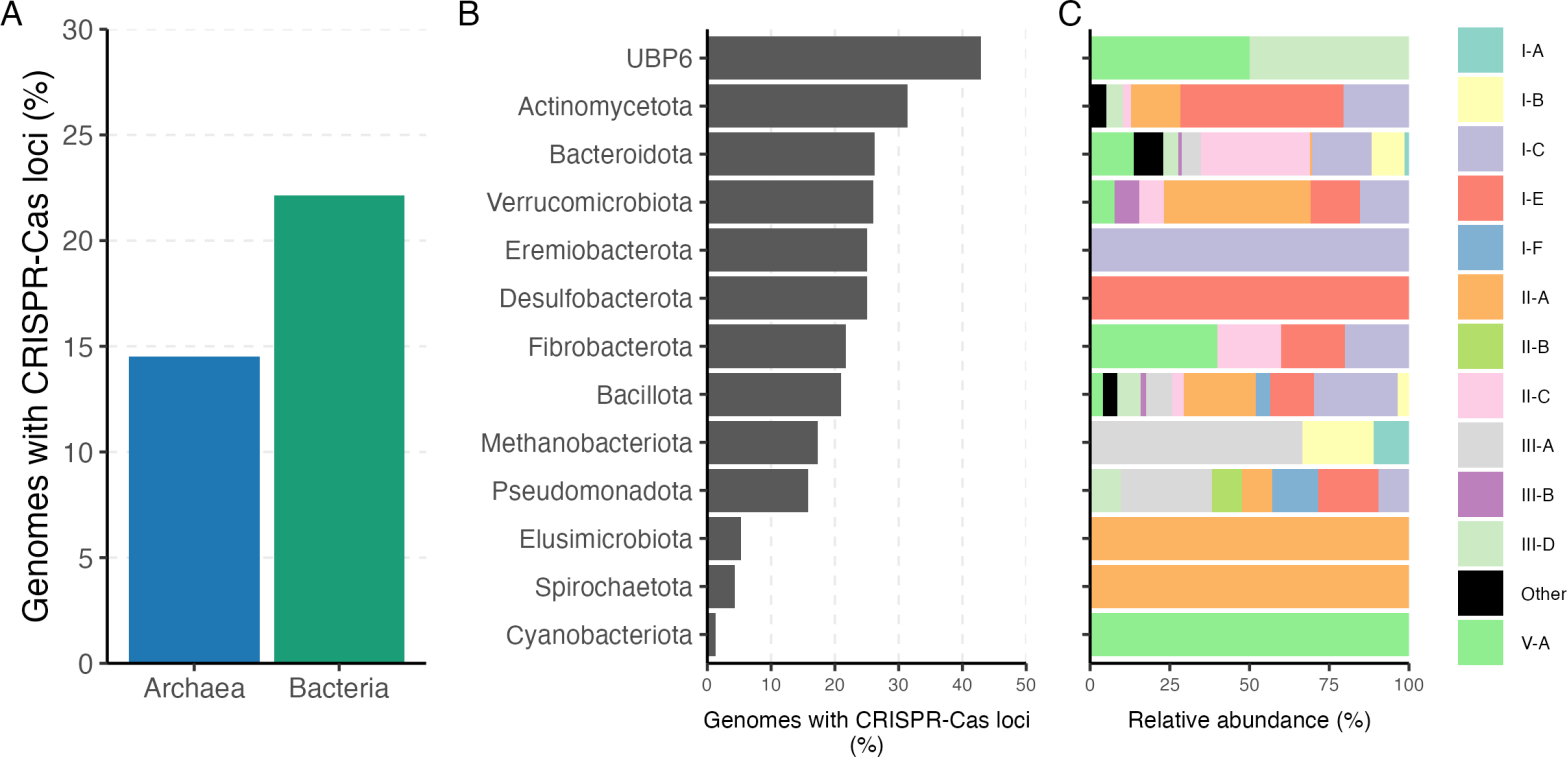
Abundance and diversity of CRISPR-Cas systems within *Bacteria* and *Archaea* in the rumen. (**A**) Percentage of genomes harboring at least one complete CRISPR-Cas loci, (**B**) percentage of genomes by phylum harboring at least one complete CRISPR-Cas loci, and (C) the relative abundance of CRISPR-Cas system types in each phylum.

Cas proteins and CRISPR arrays have been reported across complete or draft prokaryotic genomes from different environments (44, 45). However, the accurate automated prediction of CRISPR-Cas loci is challenging due to the variability in genome quality and the ambiguous prediction of the system subtypes. Therefore, we used CRISPRCASTyper to identify complete loci, and the system subtype based on both Cas genes and CRISPR repeats in the rumen genomes. In summary, 791 CRISPR-Cas complete loci were found in 668 genomes. This indicates that most of these genomes carry only one complete system. In total, 21.9% of all 3038 rumen microbiome genomes harbor at least one complete CRISPR-Cas locus. When compared, more bacterial genomes seem to carry more complete systems than archaeal genomes, 22.1% and 14.5% respectively (Fig. 6A). The phylum with the highest prevalence of CRISPR-Cas was the uncultured UBP6 (42% of genomes), followed by *Actinomycetota* (31%), *Bacteroidota* (26%) and *Verrucomicrobiota* (26%) (Fig. 6B). Interestingly, of the phyla represented by at least 50 genomes, only *Cyanobacteriota* was almost devoid of complete CRISPR-Cas. In this case, one genome out of 77 carried an array. In total, 21 CRISPR-Cas subtypes were predicted across all genomes and among them the subtype I-C was the most abundant (21.9%), followed by II-C (15.1%), II-A (13.5%) and I-E (10.3%). The number of subtypes found per phyla greatly varied, being 15 the maximum in *Bacillota* followed by 13 in *Bacteroidota*. However, in the phylum *Spirochaetota*, *Eremiobacterota*, *Elusimicrobiota*, and *Desulfobacterota* only one subtype was found in all genomes (Fig. 6C).

**Fig 7:**
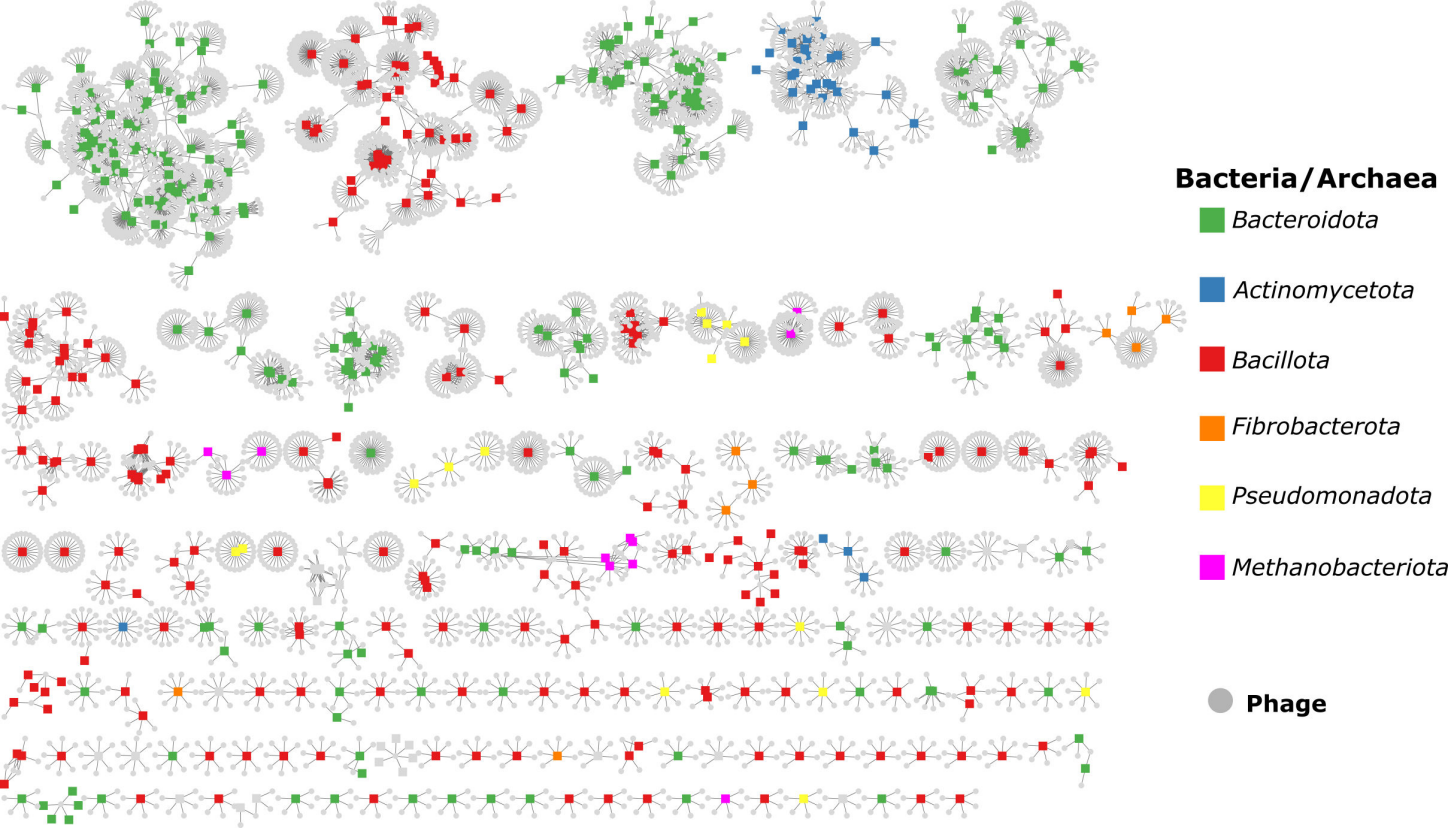
Interaction between rumen phages and their host. The host-virus interaction network shows the match between the identified CRISPR spacers from this study and species-level viral operational taxonomic units (vOTU) obtained from the global rumen virome database (RVD). Gray circles indicate a vOTU while squares represent bacterial/archaeal genomes coloured based on their phylum.

All CRISPR spacers predicted by CRISPRCASTyper were collected, including those from incomplete CRISPR-Cas locus. In total, 27,717 spacers were found in 845 genomes, ranging from one to one hundred spacers per array. All collected spacers were aligned against a rumen virome database (RVD) using blast, containing 397,180 species-level viral operational taxonomic units (vOTUs). The vOTUs were mined from 975 public rumen metagenomes, including samples from four different continents and 13 animal species. In total, 30.2% of the spacers matched against 4,986 vOTUs, which could indicate a previous history of phage-bacterial and phage-archaeal infection (Fig. 7; Table S4). Most of the interactions were found between members of the phylum *Bacillota*, *Bacteroidota* and *Actinomycetota* and different vOTUs. For example, two uncultured species from the *Firmicutes* group A have a history of interaction with 53 and 52 different vOTUs, which was the maximum found (Table S5). Similarly, the spacers of the archaea *Methanobrevibacter* and bacteria *Prevotella* matched with 49 and 39 different vOTUs, respectively. Additionally, approximately 7% of the collected phages showed intra-species host specificity, while 2.6% intra-genus host specificity (Table S6). Only 1.8% of the vOTUs that matched with the spacers have been classified as members of the *Siphoviridae* and *Myoviridae* families, while the rest remain unclassified.

## DISCUSSION

It is estimated that phages are 10-times more abundant than bacteria and their role in shaping microbial communities, influencing genetic diversity, and participating in biogeochemical cycles is a crucial aspect of the Earth’s ecosystems (46, 47). At the individual level, the lysis of the bacterial cell by a phage seen to be mainly a predatory interaction. However, at the population level, the bacteria-phage interaction can be understood as antagonistic co-evolutionary mutualism, where the continuous interaction between prey and predator generates genetic diversity that provides a mutually beneficial outcome (48). In addition, it has been hypothesized that in animal mucosal surfaces with high microbial abundance and growth rate, phages switch to the temperate life cycle and "Piggyback-the-Winner" (49). This model suggests a cooperative relationship in which the temperate phages replicate within the high-density bacterial host while preventing a closely related phage from infecting the same bacterial cell. In the rumen, phages are diverse and seem to infect the core microbiome, including *Prevotella*, *Ruminococcus*, *Fibrobacter*, *Butyrivibrio*, and *Methanobrevibacter* (13, 50). This is especially important because the arms race between phages and host can modulate the functional traits in the rumen (14). This arms race is driven by defense systems in prokaryotes and by anti-defense proteins in phages and other mobile genetic elements (MGEs) (19, 20). In the rumen we found that defense systems are diverse, mainly associated to innate immunity and dominated by RM, abortive systems and cas proteins. The high prevalence of defense systems with a broad genetic organization suggests that selective pressure has been exerted by phages and other MGEs in the rumen. However, this pressure also triggers the evolution of antidefense encoded in phages. For example, proteins that inhibit CRISPR-Cas, Gabija, Thoeris, and Hachiman systems are present in phages that infect taxonomically diverse bacterial species (20, 51, 52). The rumen is not the exception as it has been found that rumen phages carry anti-RM system, which represents a counter-defense mechanism of the most abundant prokaryotic defense (13). The emergence of different strategies to evade defense systems in phages and other MGEs could drive the rapid acquisition of diverse and novel defense systems in prokaryotes. This arms race extends even between viruses, as phages are reservoir of antiviral systems which can inhibit competing viruses (53, 54).

The high variability of defense systems and defense system families across the genomes from different environments, including the rumen, could back the “pan-immune system” model (21). This model suggests that genetically closely related strains could share defense systems via horizontal gene transfer, which indicates that the immunity could be driven by the collection of systems in the community. MGEs play a main role in the mobilization of defense systems because they often carry defense genes, even more frequently compared to chromosomes (55, 56). Moreover, defense system seen to organize in defense island, accompanied by MGEs, from which novel defense genetic architecture could emerge (22, 55). In the rumen, the pan-immunity could be driven mainly by the innate response, as CRISPR-Cas is only present in approximately 21% of the rumen genomes, but they have been shown to be important in the evolutionary dynamic between phage and their host in the rumen (14). This seems to be common also in other environments like cheese and silage-associated bacterial communities (57, 58). The genomes analysed in the present study harboring CRISPR-Cas systems showed a history of infection with phages mined from rumen samples. Some of this phages could infect not only several genomes but also different species and genera, which has been previously reported in samples from ruminants (59), the human gut (60) and the ocean. A broad host range could possibly have arisen through adaptation to different receptors by mutations, which is also considered as an advantage for the arms race without acquiring anti-defense systems (61). Such polyvalent phages can influence the rumen metabolic traits at the cellular and microbiome level. For example, our data showed that *Prevotella* and *Methanobrevibacter* with a wide repertoire of defense mechanism, involved in fiber degradation and methanogenesis, have a broad interaction with phages (62, 63). This was consistent with previous reports on the modulating role of the rumen viral community in key metabolic traits like nutrient recycling, fiber degradation and methanogenesis (14, 64). This shows that even though in low proportion the adaptive immunity can participate in the response of specific infections that can modulate the microbial and animal metabolism.

Moreover, the present study adds to the growing evidence that highlights the prevalence and significance of antiviral defense systems in prokaryotic communities. We show that defense systems in the rumen microbiome are prevalent, with almost 90% of the bacterial and archaeal genomes carrying at least one system. A similar number of defense systems has been observed within genomes from the NCBI RefSeq (24) and MAGs from soil and human gut (Fig. S5) (55). Additionally, the number of systems per genome from different environments seem to follow a binomial distribution, where most genomes carry between 2 and 5 systems. We observed a similar distribution within the families of the defense systems in the rumen. The combination of different systems can provided a broader resistance and resistance range (65, 66) and the widespread distribution of these systems suggests a crucial role in maintaining microbial diversity and ecosystem stability.

Also, our findings further reinforce the notion that defense mechanisms are diverse but just a few are dominant. Consistently, RM, Abi, and Cas are the most abundant defense system families across different genomes and environments (55, 67). At least in the rumen these three families represent approximately 70% of all defense systems. Furthermore, 28 out of the total 49 families found in the rumen were present only in 1% of the genomes. This shows that these systems are rare, but they are still present in diverse genomes. Such numbers have been observed in a collection of 21,000 RefSeq genomes, which also suggests that the diversity of antiviral systems is bigger than previously thought (24).

Different environmental and genetic factors can contribute to the selection of genes (68, 69). Previous studies have shown that bacterial and phage genome, and plasmid size seems to drive the acquisition of defense mechanisms (24, 44, 70). We also found that this is true for bacteria and archaea in the rumen, as the number of defense systems positively correlated with genome size. Genomes would tend to be larger when defense systems such as CRISPR-Cas are present due to their size, and the same could apply when multiple small defense systems are recruited in the same genome or defense islands (55). On the other hand, the number of prophages in the genome had a low or positive correlation with the number of defense systems. We were expecting a negative correlation between the number of defense systems and prophages, as more systems can provide a broader resistance, but it was not the case in our samples. However, other studies have found that the abundance of CRISPR-Cas systems can be linked with the viral abundance (71).

It is relevant to point that our study has a few limitations. Firstly, most of the used genomes in the study are product of MAG binning, which is known to poorly reconstruct closely related strains (72). This is especially important, because it could limit the detection of defense systems due to the variations of defense system composition between strains (22). Additionally, short-sequencing may not provide accurate genome-specific signals, low abundance, and could affect the sensitivity/specificity trade-off of the single-gene or multi-gene system detection specially in large CRISPR arrays (23). Secondly, the unbalanced number of isolates and MAGs shows that the rumen microbiome is still largely unculturable. In this way the description of the rumen defense system composition is mostly computational dependant. Also, isolates tend to harbor more defense system, defense system families and a higher defense system density, which can be associated with the genome completeness and low fragmentation. The accumulation of more defense mechanism in isolates compared to MAGs can be mainly associates to the methods used to recover them and not their lifestyle or role in the microbial community. However, a larger portion of isolates need to be collected to draw robust conclusion. Besides these limitations, we believe that this targeted data analysis is a starting point to expand the search for defense system in the rumen using state of the art techniques such as long-read sequencing and high throughput culturomics.

### Conclusion

Antiviral defense systems are prevalent and diverse in the rumen, but only a few are dominant, indicating that many systems are rarely present. However, the collection of systems throughout the rumen may represent a pool of mechanisms that can be shared by different members of the community. This could support the “pan-immune system” model, which appears to be common across different environments.

## Data Availability

Data processing workflow as well as other R scripts for data wrangling and visualization can be found as a Jupyter-lab notebook at https://github.com/SebasSaenz/Papers_wf. The MGnify cow rumen v1.0 MAG catalogue collection can be found at http://ftp.ebi.ac.uk/pub/databases/metagenomics/mgnify_genomes/cow-rumen/v1.0/, and the Hungate 1000 catalogue of reference genomes from the rumen microbiome is available at https://genome.jgi.doe.gov/portal/TheHunmicrobiome/TheHunmicrobiome.info.html.

## SUPPLEMENTAL MATERIAL

Supplemental tables msystems.01521-24-s0001.xlsx Tables S1-S6.

Supplemental figures msystems.01521-24-s0002.docx Figures S1-S5.

ASM does not own the copyrights to Supplemental Material that may be linked to, or accessed through, an article. The authors have granted ASM a non-exclusive, world-wide license to publish the Supplemental Material files. Please contact the corresponding author directly for reuse.
