# Supplementary material for: Antiviral defense systems in the rumen microbiome
Source: mSystems. 2025 Jan 14;10(2):e01521-24. doi: 10.1128/msystems.01521-24 (PMC11834463; doi:10.1128/msystems.01521-24)
Supplement: Supplemental figures — Figures S1-S5. [file msystems.01521-24-s0002.docx]

**Antiviral defence systems in the rumen microbiome**

**Johan S. Sáenz^1,2,*^, Bibiana Rios-Galicia^1,2^ & Jana Seifert^1,2^**

^1^Institute of Animal Science, University of Hohenheim, Emil-Wolff-Str. 6-10, 70593 Stuttgart, Germany

^2^HoLMiR—Hohenheim Center for Livestock Microbiome Research, University of Hohenheim, Leonore-Blosser-Reisen Weg 3, 70593 Stuttgart, Germany

*Corresponding author: [johan.saenzmedina@uni-hohenheim.de](mailto:johan.saenzmedina@uni-hohenheim.de)

**Supplementary material**


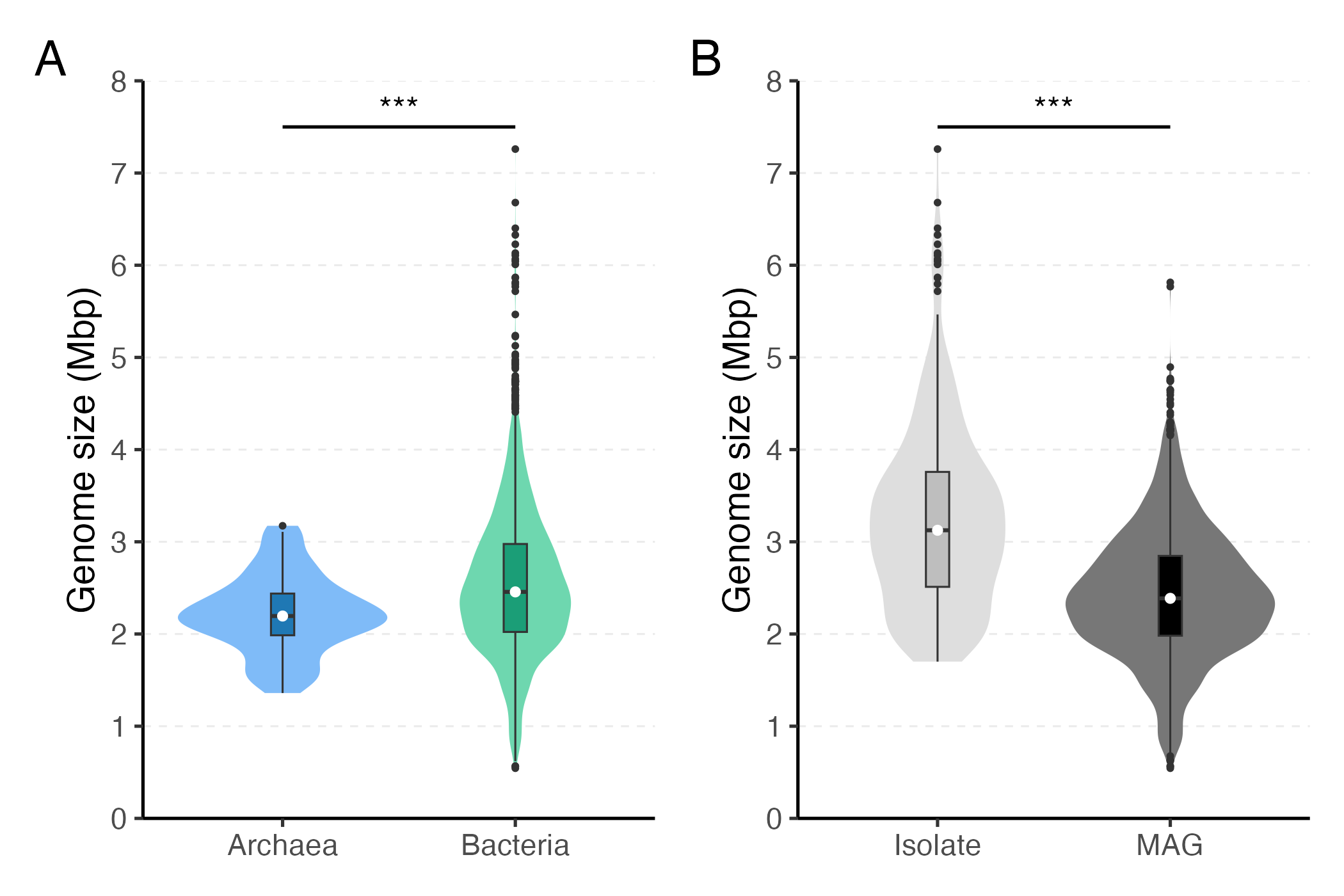


**Figure S1. Genome size.** Comparison of the genome size between the A) archaeal and bacterial genomes and B) isolates and MAGs. Kruskall-Wallis test *** < 0.0005


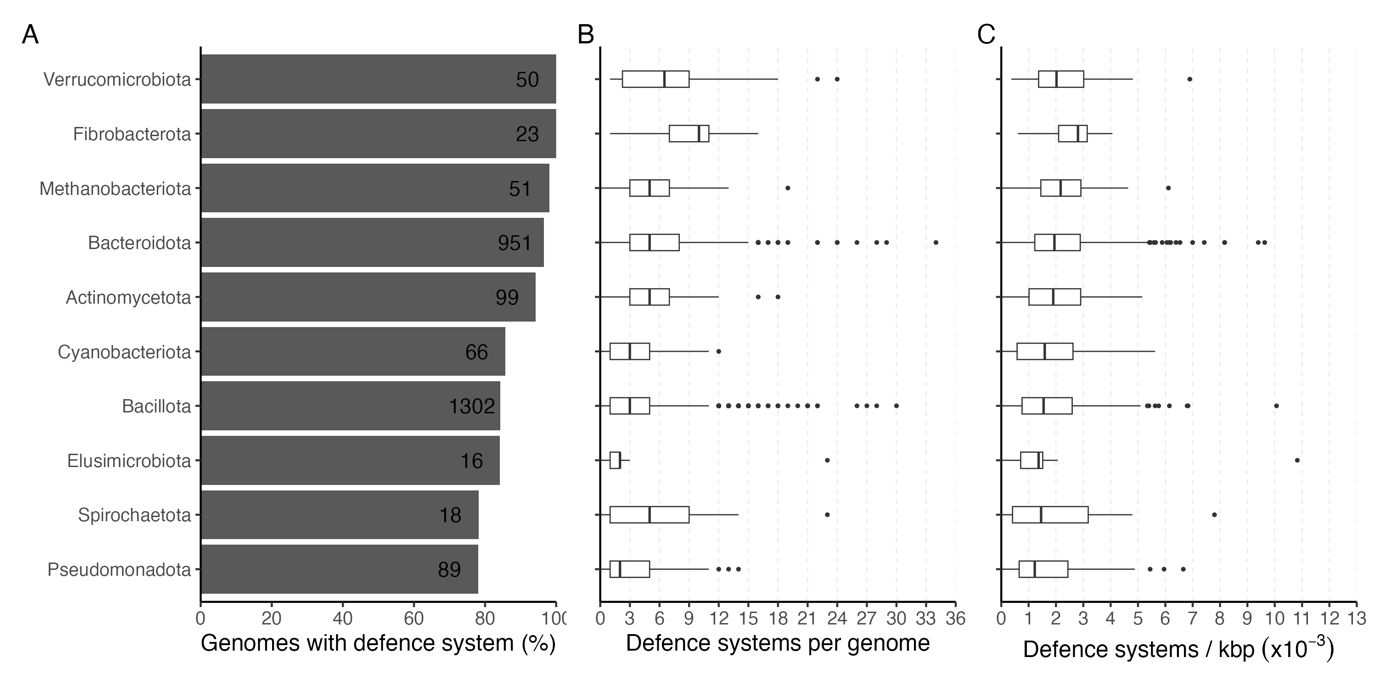


**Figure S2.** Prevalence, abundance and density (per genome per kbp) of defence systems across the rumen phyla. Phyla with more than 10 genomes in the dataset were included.


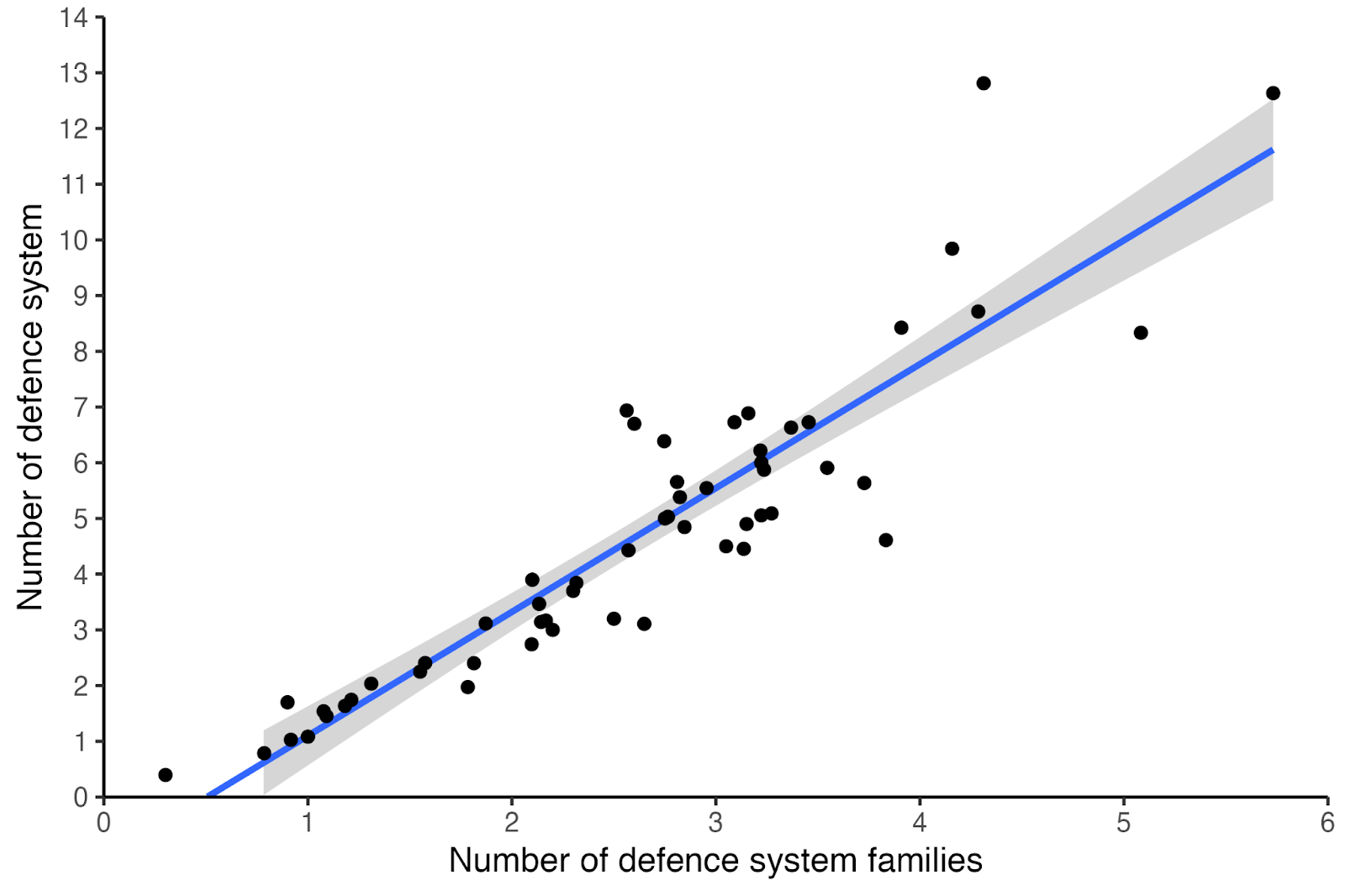


**Figure S3. Correlation between the number of defence systems and defence system families harboured per genome.** Each dot represents the average number of defence system families and defence systems found in the most prevalent genera of the dataset. Only the genera represented by more than 10 genomes were selected (n=57).


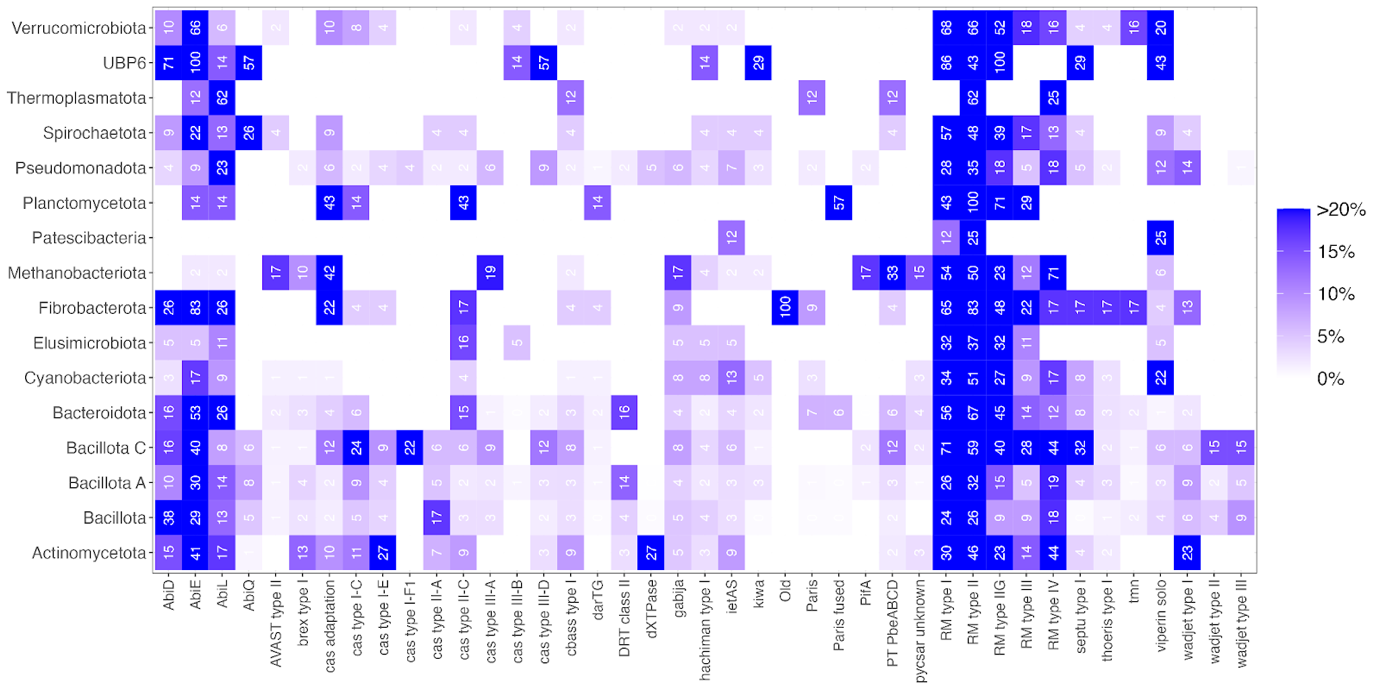


**Figure S4. Prevalence of defence mechanisms across different bacterial and archaeal phyla in the cow-rumen microbiome.** The relative abundance of the different system is shown with the blue colour


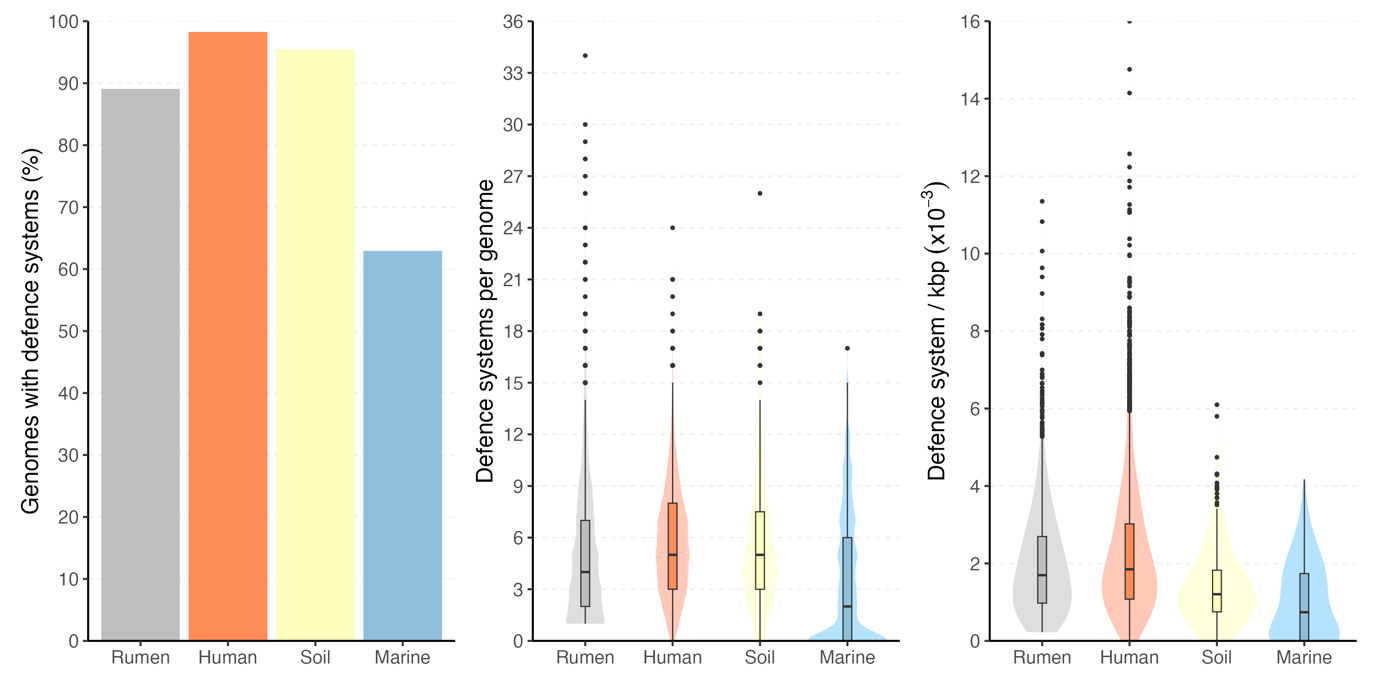


**Figure S5.** Comparison of defence system prevalence, abundance and density across several environments. The data from human, soil and marine was obtained from (Beavogui et al. 2024)

Beavogui, Angelina, Auriane Lacroix, Nicolas Wiart, Julie Poulain, Tom O. Delmont, Lucas Paoli, Patrick Wincker, and Pedro H. Oliveira. 2024. “The Defensome of Complex Bacterial Communities.” *Nature Communications* 15 (1): 2146
